# COVID-19 Vaccine Willingness and Related Factors Among Health Care Workers in 3 Southeast Asian Jurisdictions

**DOI:** 10.1001/jamanetworkopen.2022.28061

**Published:** 2022-08-22

**Authors:** Eliza Lai-yi Wong, Hong Qiu, Wai Tong Chien, Jojo Cho-lee Wong, Hom Nath Chalise, Huong Thi-xuan Hoang, Hong Trang Nguyen, Paul Kay-sheung Chan, Martin Chi-sang Wong, Annie Wai-ling Cheung, Eng-kiong Yeoh

**Affiliations:** 1 Centre for Health Systems and Policy Research, JC School of Public Health and Primary Care, Faculty of Medicine, The Chinese University of Hong Kong, Hong Kong; 2 The Nethersole School of Nursing, Faculty of Medicine, The Chinese University of Hong Kong, Hong Kong; 3 Department of Public Health, Nobel College, Affiliated to Pokhara University, Sinamangal, Kathmandu 47000, Nepal; 4 Phenikaa University, Hanoi, Vietnam; 5 Department of Microbiology, JC School of Public Health and Primary Care, Faculty of Medicine, Chinese University of Hong Kong, Hong Kong; 6 JC School of Public Health and Primary Care, Faculty of Medicine, Chinese University of Hong Kong, Hong Kong

## Abstract

**Importance:**

COVID-19 vaccine hesitancy is widespread and may lead to refusal or delay of vaccination, eventually reducing the overall vaccination coverage rate and vaccine effectiveness. Willingness to receive COVID-19 vaccination among health care workers (HCWs) is diverse across different jurisdictions.

**Objective:**

To assess the COVID-19 vaccine willingness among HCWs in 3 Southeast Asian jurisdictions in the context of pandemic severity and vaccination policy.

**Design, Setting, and Participants:**

A cross-sectional online survey was conducted among frontline HCWs in Hong Kong, Nepal, and Vietnam from May to November 2021. Eligible participants were nurses and doctors aged 18 and older, working in public or private health care settings on a full-time or part-time basis.

**Exposures:**

The COVID-19 pandemic and vaccination policy.

**Main Outcomes and Measures:**

COVID-19 vaccination willingness was defined as HCW willingness toward receiving the COVID-19 vaccine in full course or the first dose of the vaccine, and willingness to take the second dose. Information on sociodemographic characteristics, the history of seasonal influenza vaccination, attitudes toward vaccination, and opinions on strategies associated with vaccination uptake from the study participants.

**Results:**

Among the 3396 eligible doctors and nurses who participated in the survey, 2834 (83.4%) were from Hong Kong, 328 (9.7%) were from Nepal, and 234 (6.9%) were from Vietnam. Most respondents were female (76.2% [2589 ]), aged 30 to 39 years (31.2% [1058]), and nurse HCWs (77.6% [2636]); the response rates were 11% (2834 of 25 000) in Hong Kong, 36% (328 of 900) in Nepal, and 13% (234 of 1800) in Vietnam. Overall, the prevalence rate of willingness to take the COVID-19 vaccine was highest in Nepal (95.4% [313 of 328]), followed by Vietnam (90.6% [212 of 234]), and lowest in Hong Kong (54.4% [1542 of 2834]), relating to their different attitudes and opinions toward the COVID-19 vaccination, and the pandemic severity and vaccination policy in the 3 jurisdictions. Doctors were more willing to take COVID-19 vaccination than nurses (odds ratio, 5.28; 95% CI, 3.96-7.04). Older age (odds ratios, 1.39-3.70), male gender (odds ratio, 1.41; 95% CI, 1.11-1.75), higher educational level (odds ratio, 1.48; 95% CI, 1.17-1.87), and having seasonal influenza vaccination uptake history (odds ratio, 2.15; 95% CI, 1.82-2.54) were found to be associated with increased willingness. Choice of vaccination brand with adequate information, immunity passport, time off from work for vaccination and subsidy for travel to inconvenient vaccination centers were considered as strategies to enhance vaccine willingness.

**Conclusions and Relevance:**

In this survey study, vaccination unwillingness existed among HCWs in Southeast Asian regions, especially in Hong Kong. The findings of this study may have utility in the formulation of vaccination promotion strategies such as vaccination incentives. Attitudes toward vaccination in HCWs might be examples for the general population; however, changes over time should be further investigated.

## Key Points

**Question:**

What are the COVID-19 vaccine willingness rates among the health care workers (HCWs) in 3 Southeast Asian jurisdictions in the context of pandemic severity and vaccination policies?

**Findings:**

In this survey study including responses from 3396 doctors and nurses , willingness to take the COVID-19 vaccine was highest in Nepal, followed by Vietnam, and lowest in Hong Kong, which may be associated with the pandemic severity and vaccination policy in the 3 jurisdictions. Type of HCW (doctor), older age, male gender, higher educational level, and having seasonal influenza vaccination history were found to be factors associated with vaccination willingness.

**Meaning:**

The findings of this study may have utility in informing future public health policies and strategies to promote vaccine acceptance during pandemics.

## Teaser

This survey study identifies and compares rates of COVID-19 vaccine willingness among health care workers in Hong Kong, Nepal, and Vietnam and associated factors in the context of authorized COVID-19 vaccine initiatives.

## Introduction

The COVID-19 pandemic is a global challenge, and newly developed COVID-19 vaccines^1^ can reduce risk of febrile symptoms^2^ and protect those most vulnerable from serious consequences.^3,4^ However, because COVID-19 vaccine is novel, COVID-19 vaccine hesitancy exists widely and may lead to refusal or delay of vaccination, eventually reducing the coverage rate and effectiveness.^5,6,7^

Frontline health care workers (HCWs) are at a higher risk of exposure to the virus when performing diagnoses and treatments on patients and thus have a higher risk of infection.^8,9^ A study in the UK and the US showed that frontline HCWs have a 10.6-fold increased risk of positive tests compared with the general population.^9^ Prevention of infection among HCWs has a protective effect on both patients and medical staff. A review of 38 cross-sectional studies worldwide indicated that there were varying levels of willingness to receive the COVID-19 vaccination, with a lower acceptance rate (65.7%) in HCWs than in the general population (81.7%).^10^ Another study observed COVID-19 vaccination hesitancy rate in HCWs widely ranged from 4.3% to 72%, with an average of 22.5% across included studies.^11^

Although COVID-19 vaccine willingness and its associated factors have been widely discussed in the literature, they were mainly studied in single jurisdictions with varying results.^12,13^ The issues of vaccine willingness require a delicate ethical balancing of individual liberties, social responsibilities to protect public health, and employment obligations in the context of macroenvironments. Hong Kong, Nepal, and Vietnam are all located in Southeast Asia but were categorized into high-income (Hong Kong) and middle-income (Nepal and Vietnam) countries according to economic status. The different health care systems, cultures, and social-economic statuses of these jurisdictions could potentially mean varying attitudes and rationales toward COVID-19 vaccination in populations. To boost injection rates, different policies or initiatives were introduced during the survey period. Frontline HCWs is one of the priority groups eligible for free vaccination programs in all 3 jurisdictions. To further encourage vaccination uptake among HCW’s, the Vietnamese government placed family members of HCWs onto the priority list to receive free vaccines, while HCWs in Hong Kong were entitled to a day of vaccination leave for each vaccination dose received. With regard to the choice of COVID-19 vaccination, Hong Kong citizens had 2, Vietnam had 5, and Nepal had 1. A regular COVID-19 test policy has been applied to HCWs in 3 jurisdictions. Details are shown in Table 1.^14,15,16,17^ This study aimed to identify and compare COVID-19 vaccine willingness among HCWs in these 3 jurisdictions and investigate their associated factors in the context of authorized COVID-19 vaccine initiatives.

**Table 1.** Macroenvironment in 3 Jurisdictions

Variable	Hong Kong^14^	Nepal^15^	Vietnam^16^
**COVID-19 vaccine initiatives**			
Priority group	HCW is one of priority to access to vaccine	HCW is one of priority to access to vaccine	HCW is one of priority to access to vaccine
Payment	Free	Free	Free
Policy	Not compulsory but mandatory vaccine or free regular PCR testing	Not compulsory, free PCR testing for those with symptoms no matter vaccinated or non-vaccinated	Neither compulsory nor mandatory, but majority hospitals conducted free PCR test for HCW without vaccination
Choice	2 choices: Sinovac-CoronaVac, BioNTech	1 choice: Moderna	5 choices: Sinovac-CoronaVac, BioNTech, Moderna, Astra-Zeneca, Sputnik-V
Incentive	Vaccination leave	No	Family of HCW is one of priority to access vaccine
**COVID-19 Pandemic** ^17^ ^a^			
Averaged daily incident rate per 100 000 population	0.03	3.63	8.13
Case mortality rate, %	0.81	1.37	2.24

Abbreviations: HCW, health care worker; PCR, polymerase chain reaction.

^a^ Time of survey for Hong Kong was fourth wave (May 11 to June 23, 2021); for Nepal was third wave (August 10 to November 7, 2021); and for Vietnam was fourth wave (July 12 to November 20, 2021).

## Methods

### Study Design and Participants

A multicenter online self-administered cross-sectional survey in 2021 was conducted among HCWs in Hong Kong from May 11 to June 23 (the fourth wave); in Nepal from August 10 to November 7 (the third wave); and in Vietnam from July 12 to November 20 (the fourth wave). Two rounds of email invitations were disseminated to the HCWs through the health care professional bodies and unions in Hong Kong and to major hospitals and a network of HCW voluntary consult for COVID-19 patients in Vietnam and Nepal for the recruitment. The online survey platform Qualtrics (Qualtrics LLC) was used to create the questionnaire (eMethods in the Supplement), distribute and store the collected responses. Eligible participants were nurses or doctors aged 18 or older, working in either public or private health care settings on a full-time or part-time basis. Informed consent was obtained from each respondent online before taking the survey. The study followed the American Association for Public Opinion Research (AAPOR) reporting guideline and was approved by the Joint Chinese University of Hong Kong–New Territories East Cluster Clinical Research Ethics Committee.

### Data Collection

A structured questionnaire was developed based on international and local studies.^8,18,19,20^ The questions related to the status of COVID-19 vaccination of the respondents, their attitudes or opinions toward the COVID-19 vaccination, their perceived challenges and concerns; information on the reasons for not receiving vaccination and strategies to encourage vaccine uptake were collected. Information on sociodemographic characteristics such as age, gender, employment status, and education level, and their history of seasonal influenza vaccination were also collected.

#### COVID-19 Vaccination Status and Willingness

COVID-19 vaccination status was assessed through the question, “Have you received COVID-19 vaccination?” The responses consisted of 4 categories: “No vaccination,” “Yes, in full course,” “First dose only, will complete,” and “First dose only, will not complete.” We defined willingness to accept COVID-19 vaccination as taking the COVID-19 vaccine in a full course or the first dose of the vaccine and being willing to take the second dose. Participants who had not taken COVID-19 vaccination and those who have received the first dose of the vaccine but would not complete the vaccine schedule were defined as people with COVID-19 vaccine unwillingness or hesitancy.

#### Attitudes and Opinions Toward COVID-19 Vaccination

Questions about attitudes included the concerns on the effectiveness of the COVID-19 vaccine, availability of different types of vaccine, and necessity of advice from health care professional or comprehensive vaccine information, along with the opinions on the mandatory vaccination applied for different groups of population. The attitudes toward the importance of receiving COVID-19 vaccination to safeguard health, strengthen infection control in the working environment, and achieve the herd immunity were also collected. All these statements had response as “agree” and “disagree.”

#### Reasons for Vaccination Unwillingness and Strategies to Encourage Vaccine Uptake

Participants who did not take the COVID-19 vaccine or would not take the second dose, were asked to provide their opinions on vaccine unwillingness, including their concerns about the adverse effects and safety of the vaccines, vaccine manufacturers or their places of origin, and effectiveness of the vaccine and advice from the government. Questions about the strategies in encouraging vaccine uptake included “transportation allowance,” “authorizing paid absence on the day of vaccination and the day after,” and “implementation of ‘immunity passport’ for traveling purpose.” These statements were rated on a scale with scores ranging from 0 to 10, with higher scores indicating that participants reported a greater effect of the cause.

### Statistical Analysis

Descriptive information of COVID-19 vaccination status, willingness, and the attitudes and opinions toward COVID-19 vaccination was reported. The χ^2^ test was used to compare the proportion of each of the sociodemographic characteristics, vaccine willingness, agreement for each statement of attitude, and opinion of HCWs toward the COVID-19 vaccination across the 3 study jurisdictions.

Binary logistic regression was performed to identify variables associated with COVID-19 vaccine willingness. First, we used univariate logistic regression to examine the variables associated with vaccine willingness and provide the crude odds ratio (OR) and 95% CIs. Then we included all variables of interest in the model and applied a backward stepwise multivariate logistic regression to identify statistically factors associated with willingness after adjusting the confounding from each other.

Reasons for unwillingness and strategies encouraging vaccine uptake were measured by a scale ranging from 0 to 10 for a subgroup of participants who did not take the COVID-19 vaccine and those who would not take the second dose of the vaccine. We used box plots to present the distribution of scores and compare the median scores in Hong Kong with those of Nepal and Vietnam using the nonparametric test.

The significance test was 2-sided. To control for the problem of false-positive rates induced by multiple significance tests, Bonferroni correction was applied by setting the type I error as 0.05 divided by the number of tests.^21^ We had 3 comparisons among the 3 jurisdictions, therefore, a *P* < .02 (*P* < .05 divided by 3 and rounded) was considered to be statistically significant. Data processing and analyses were conducted in R version 4.1.2 with Epi and ggplot2 packages (R Foundation for Statistical Computing).

## Results

### Demographic Characteristics

Among the 3396 eligible doctors and nurses who participated in the survey, 2834 were from Hong Kong (83.4%), 328 were from Nepal (9.7%), and 234 were from Vietnam (6.9%). Most respondents were female (76.2% [2589]), aged 30 to 39 years (31.2% [1058]), and nurse HCW (77.6% [2636]) with the response rates were 11% (2834 of 25 000) in Hong Kong, 36% (328 of 900) in Nepal, and 13% (234 of 1800) in Vietnam. The sociodemographic characteristics of the participants in the 3 jurisdictions are summarized in Table 2. The proportion aged younger than 40 years old was highest among the participants from Vietnam (76.5% [179 of 234]), followed by Nepal (58.2% [191 of 328]) and Hong Kong (44.0% [1248 of 2834]). More participants were female in Hong Kong (78.1% [2213 of 2834]), in Vietnam (70.5% [165 of 234]), and in Nepal (64.3% [211 of 328]). Most respondents were full-time employees (87.4% [2967 of 3396]) and had a bachelor’s degree or above (81.2% [2756 of 3396]). Approximately one-half of the participants took the seasonal influenza vaccinations in the last year (52.3% [1776 of 3396]), with the highest in Hong Kong (55.3% [1567 of 2834]), followed by Nepal (45.1% [148 of 328]), and lowest in Vietnam (26.1% [61 of 234]).

**Table 2.** Sociodemographic Characteristics and Acceptance of COVID-19 Vaccine in 3396 Health Care Workers in Hong Kong, Nepal, and Vietnam^a^

Characteristics	No. (%)				*P* value^b^		
	Total (N = 3396)	Hong Kong (n = 2834)	Nepal (n = 328)	Vietnam (n = 234)	Hong Kong vs Nepal	Hong Kong vs Vietnam	Nepal vs Vietnam
Type of health care worker							
Nurse	2636 (77.6)	2257 (79.6)	193 (58.8)	186 (79.5)	<.001	>.99	<.001
Doctor	760 (22.4)	577 (20.4)	135 (41.2)	48 (20.5)			
Age group, y							
18-29	560 (16.5)	405 (14.3)	97 (29.6)	58 (24.8)	<.001	<.001	<.001
30-39	1058 (31.2)	843 (29.7)	94 (28.7)	121 (51.7)			
40-49	834 (24.6)	722 (25.5)	68 (20.7)	44 (18.8)			
≥50	928 (27.3)	848 (29.9)	69 (21.0)	11 (4.7)			
Gender							
Male	788 (23.2)	602 (21.2)	117 (35.7)	69 (29.5)	<.001	.005	.15
Female	2589 (76.2)	2213 (78.1)	211 (64.3)	165 (70.5)			
Employment							
Full-time	2967 (87.4)	2515 (88.7)	246 (75.0)	206 (88.0)	<.001	.82	<.001
Part-time	429 (12.6)	319 (11.3)	82 (25.0)	28 (12.0)			
Education							
Diploma or below	624 (18.4)	541 (19.1)	34 (10.4)	49 (20.9)	<.001	.63	<.001
Bachelor’s degree	1596 (47.0)	1366 (48.2)	114 (34.8)	116 (49.6)			
Master’s or doctoral degree	1160 (34.2)	911 (32.1)	180 (54.9)	69 (29.5)			
Seasonal influenza vaccination last year	1776 (52.3)	1567 (55.3)	148 (45.1)	61 (26.1)	<.001	<.001	<.001
COVID-19 vaccination at survey^c^							
No	1309 (38.5)	1277 (45.1)	11 (3.4)	21 (9.0)	<.001	<.001	.008
Yes, full course (2 doses)	1638 (48.2)	1212 (42.8)	289 (88.1)	137 (58.5)			
First dose only							
Will complete	429 (12.6)	330 (11.6)	24 (7.3)	75 (32.1)			
Will not complete	20 (0.6)	15 (0.5)	4 (1.2)	1 (0.4)			
COVID-19 vaccination willingness^d^							
Yes	2067 (60.9)	1542 (54.4)	313 (95.4)	212 (90.6)	<.001	<.001	.035
No	1329 (39.1)	1292 (45.6)	15 (4.6)	22 (9.4)			

^a^ Data are presented as the number of the participants in each category (N), together with the column percentage (%); 19 participants from Hong Kong missed providing information on gender, and 16 participants from Hong Kong missed providing information on age group and educational level.

^b^ *P* values are obtained from the χ^2^ test. Bonferroni correction was applied and a *P* < .02 (*P* < .05 divided by 3) was considered statistically significant.

^c^ Comparison of the Yes or No Covid-19 vaccination status among the 3 regions.

^d^ COVID-19 vaccination willingness is defined as taking the COVID-19 vaccine in full course or taking the first dose of the vaccine and willingness to take the second dose.

#### COVID-19 Vaccination Willingness

COVID-19 vaccination willingness among the HCWs in Nepal was high at 95.4% (313 of 328) and in Vietnam at 90.6% (212 of 234). Only 54.4% (1542 of 2834) in Hong Kong were willing to take COVID-19 vaccination (Table 2).

#### Attitudes Toward COVID-19 Vaccination

Attitudes and opinions of HCWs toward COVID-19 vaccination among these 3 jurisdictions also varied greatly (Table 3). In Vietnam, 95.7% (224 of 234) of HCWs agreed that “Vaccination is the most effective,” and 93.6% (219 of 234) agreed that “More vaccines are made available,” and 95.3% (223 of 234) agreed that “Advice should be made by health care professionals,” which were higher than the proportions in Hong Kong (74.6% [2114 of 2834]) and Nepal (72.9% [239 of 328]). In Hong Kong, 75.9% (2150 of 2834) of HCWs indicated “Lack of comprehensive information makes them hesitate,” which was higher than that in Vietnam (62.0% [145 of 234]) and Nepal (48.5% [159 of 328]). A greater proportion of HCWs in Nepal and Vietnam than in Hong Kong agreed that mandatory vaccination should be applied to workers in health care or residential care (96.0% [315 of 328] in Nepal; 96.6% [226 of 234] in Vietnam; and 44.1% [1251 of 2834] in Hong Kong), service industry (95.7% [314 of 328] in Nepal; 97.9% [229 of 234] in Vietnam; and 48.3% [1368 of 2834] in Hong Kong), community services (94.2% [309 of 328] in Nepal; 97.0% [227 of 234] in Vietnam; and 50.8% [1440 of 2834] in Hong Kong), and teachers (91.5 [300 of 328] in Nepal; 96.6% [226 of 234] in Vietnam; and 47.5% [1347 of 2834] in Hong Kong). The percentage of HCWs in Vietnam were highest and the percentages of HCWs in Hong Kong were lowest in agreeing with the importance of receiving COVID-19 vaccination to safeguard one’s own health (99.1% [232 of 234] for Vietnam and 88.0% [2488 of 2834] for Hong Kong), health of family and friends (99.6% [233 of 234] for Vietnam and 88.5% [2503 of 2834] for Hong Kong), strengthen infection control in their working environment (95.7% [224 of 234] for Vietnam and 85.5% [2418 of 2834] for Hong Kong), and achieve the herd immunity (98.7% [231 of 234] for Vietnam and 85.9% [2427 of 2834] for Hong Kong).

**Table 3.** Attitudes and Opinions of 3396 Health Care Workers Toward the COVID-19 Vaccination in Hong Kong, Nepal, and Vietnam^a^

Agreed statement	No. (%)				*P* value^b^		
	Total (N = 3396)	Hong Kong (n = 2834)	Nepal (n = 328)	Vietnam (n = 234)	Hong Kong vs Nepal	Hong Kong vs Vietnam	Nepal vs Vietnam
Vaccination is the most effective	2641 (77.8)	2151 (75.9)	266 (81.1)	224 (95.7)	.04	<.001	<.001
More vaccines are made available	3062 (90.2)	2579 (91.0)	264 (80.5)	219 (93.6)	<.001	.22	<.001
Advice should be made by health care professionals	2576 (75.9)	2114 (74.6)	239 (72.9)	223 (95.3)	.54	<.001	<.001
Lack of comprehensive information makes me hesitate	2454 (72.3)	2150 (75.9)	159 (48.5)	145 (62.0)	<.001	<.001	.002
Government should provide only one vaccine	585 (17.2)	364 (12.8)	150 (45.7)	71 (30.3)	<.001	<.001	<.001
Given in good health, mandatory vaccination should be applied for							
Healthcare or residential care workers	1792 (52.8)	1251 (44.1)	315 (96.0)	226 (96.6)	<.001	<.001	.91
Workers in service industry	1911 (56.3)	1368 (48.3)	314 (95.7)	229 (97.9)	<.001	<.001	.25
Necessary community services	1976 (58.2)	1440 (50.8)	309 (94.2)	227 (97.0)	<.001	<.001	.18
Teachers	1873 (55.2)	1347 (47.5)	300 (91.5)	226 (96.6)	<.001	<.001	.02
Importance of receiving COVID-19 vaccination in the following aspects							
To safeguard your own health	3013 (88.9)	2488 (88.0)	293 (89.3)	232 (99.1)	.54	<.001	<.001
To safeguard the health of family and friends	3038 (89.6)	2503 (88.5)	302 (92.1)	233 (99.6)	.07	<.001	<.001
To strengthen infection control in the working environment	2946 (86.9)	2418 (85.5)	304 (92.7)	224 (95.7)	<.001	<.001	.19
To achieve herd immunity	2960 (87.3)	2427 (85.9)	302 (92.1)	231 (98.7)	.002	<.001	<.001

^a^ Data are presented as the number of the participants who agreed with the statement (N), together with the column percentage (%).

^b^ *P* values are obtained from the χ^2^ test. Bonferroni correction was applied and a *P* < .02 (*P* < .05 divided by 3) was considered statistically significant.

#### Possible Factors Associated With COVID-19 Vaccination Willingness

The factors associated with vaccination willingness among the HCWs were identified in the multivariate logistic regression model (Table 4). The HCWs from Nepal and Vietnam were significantly more willing to take COVID-19 vaccination than Hong Kong, with an OR of 24.12 (95% CI, 13.96-41.67) for Nepal and 17.81 (95% CI, 11.15-28.45) for Vietnam. Doctors were more willing than nurses, with an OR of 5.28 (95% CI, 3.96-7.04), while older participants had higher COVID-19 vaccine willingness. Compared with the group aged 18 to 29 years, the OR for willingness was 1.39 (95% CI, 1.07-1.79) for the group aged 30 to 39 years, 3.14 (95% CI, 2.40-4.11) for the group aged 40 to 49 years, and 3.70 (95% CI, 2.84-4.83) for the group aged 50 years or older. Female participants had 30% lower willingness than males. HCWs with higher educational levels were more likely to take COVID-19 vaccination compared with the educational level of diploma or lower level, master's or doctoral degree holders had an OR of 1.5 (95% CI, 1.2-1.9). Participants who received seasonal influenza vaccination in the previous year were more willing to receive COVID-19 vaccination (OR, 2.15; CI, 1.82-2.54).

**Table 4.** Identifying Factors Associated With COVID-19 Vaccine Willingness Among 3396 Healthcare Workers in 3 Jurisdictions Merged^a^

Factors	No. (%)		OR (95% CI)^b^	
	Willingness (n = 2067)	Unwillingness (n = 1329)	Univariate model	Backward stepwise multivariate model
Region				
Hong Kong	1542 (74.6)	1292 (97.2)	1 [Reference]	1 [Reference]
Nepal	313 (15.1)	15 (1.1)	17.48 (10.36-29.50)	24.12 (13.96-41.67)
Vietnam	212 (10.3)	22 (1.7)	8.07 (5.17-12.60)	17.81 (11.15-28.45)
Type of health care worker				
Nurse	1386 (67.1)	1250 (94.1)	1 [Reference]	1 [Reference]
Doctor	681 (32.9)	79 (5.9)	7.77 (6.08-9.93)	5.28 (3.96-7.04)
Age group, y				
18-29	273 (13.3)	287 (21.7)	1 [Reference]	1 [Reference]
30-39	556 (27.0)	502 (37.9)	1.16 (0.95-1.43)	1.39 (1.07-1.79)
40-49	566 (27.5)	268 (20.2)	2.22 (1.78-2.77)	3.14 (2.40-4.11)
≥50	661 (32.1)	267 (20.2)	2.60 (2.09-3.24)	3.70 (2.84-4.83)
Gender				
Male	620 (30.2)	168 (12.7)	1 [Reference]	1 [Reference]
Female	1436 (69.8)	1153 (87.3)	0.34 (0.28-0.41)	0.71 (0.57-0.90)
Employment				
Full-time	1794 (86.8)	1173 (88.3)	1 [Reference]	NA
Part-time	273 (13.2)	156 (11.7)	1.14 (0.93-1.41)	NA
Educational level				
Diploma or below	281 (13.7)	343 (25.9)	1 [Reference]	1 [Reference]
Bachelor’s degree	973 (47.3)	623 (47.1)	1.91 (1.58-2.30)	1.16 (0.93-1.44)
Master’s or doctoral degree	802 (39.0)	358 (27.0)	2.73 (2.24-3.34)	1.48 (1.17-1.87)
Seasonal influenza vaccination last year	1238 (59.9)	538 (40.5)	2.20 (1.91-2.53)	2.15 (1.82-2.54)

Abbreviations: NA, not applicable; OR, odds ratio.

^a^ COVID-19 vaccination willingness is defined as taking the COVID-19 vaccine in full course or taking the first dose of the vaccine and will take the second dose.

^b^ OR (95% CI) was estimated from the binary logistic regression; 19 participants with missing information on gender and 16 participants with missing information on age group and educational level were excluded from the regression model.

#### Reasons for Vaccination Unwillingness and Strategies to Encourage COVID-19 Vaccine Uptake

Reasons and opinions for unwillingness and strategies encouraging vaccine uptake in the 3 jurisdictions are shown in the Figure. Among the subsample of 751 respondents who were unwilling to take the vaccine, the most common probable reasons in 3 jurisdictions were “want to wait for a better vaccine,” “aware of the instances of death or serious adverse effects following vaccination,” “concerned of the vaccine manufacturer or its place of origin,” and “confused by different information of the vaccine” (Figure, A). Agreement with “don't believe in the advice of the government” was significantly higher in Hong Kong (Figure, A) whereas the 3 statements: “vaccination venues are inconvenient,” “I don’t have a choice in vaccine update,” and “to provide transportation allowance” were significantly higher in Vietnam and Nepal (Figure, B and C).

**Figure.**
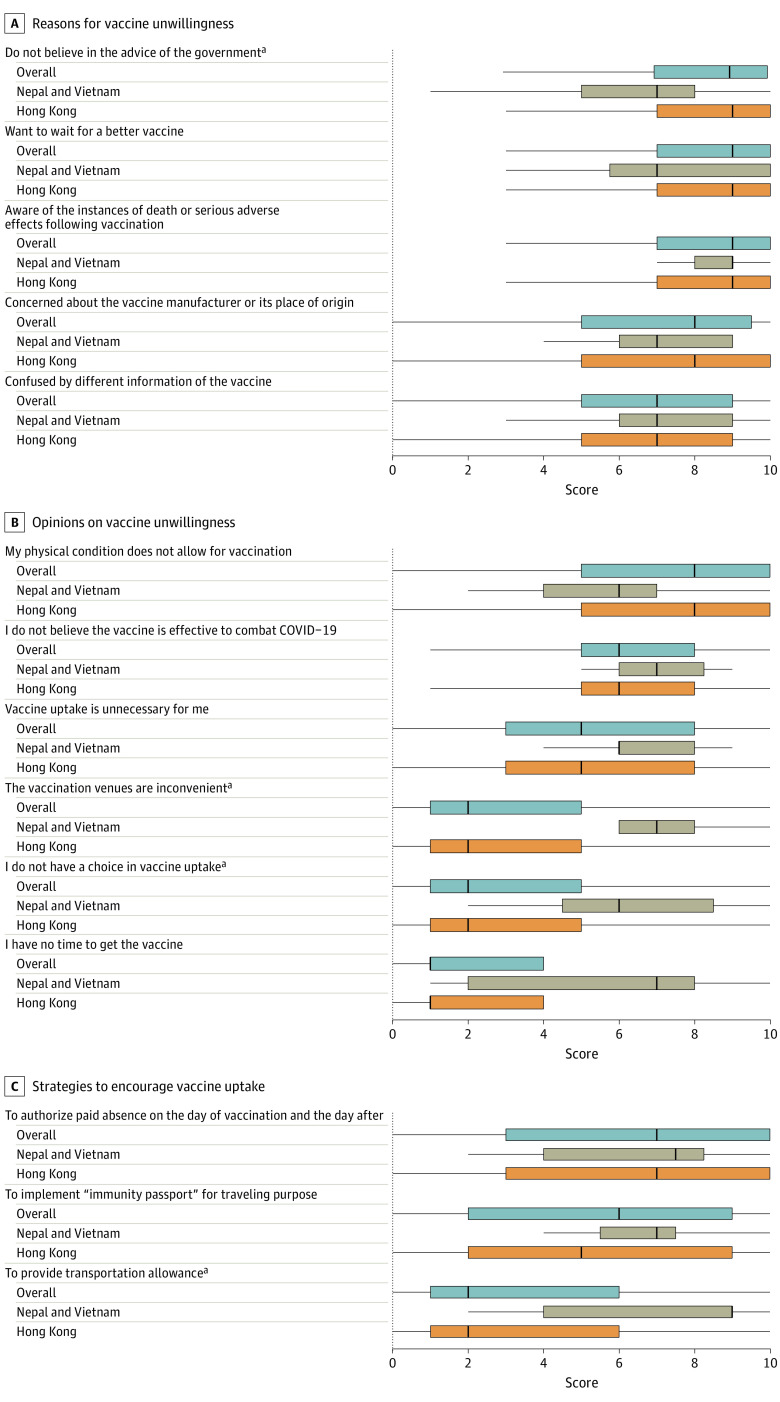
Reasons and Opinions for Vaccine Unwillingness and Strategies to Encourage Vaccine Uptake Among 751 HCWs in Hong Kong, Nepal, and Vietnam The left and right line of each box plot is the IQR; the bold line, the median. ^a^In the label of y-axis, *P* < .05 from the nonparametric 2-sample test when comparing the median score in Hong Kong with Nepal and Vietnam combined.

## Discussion

In 3 Southeast Asian jurisdictions, we observed the highest COVID-19 vaccine willingness among HCWs in Nepal, followed by those in Vietnam and Hong Kong, corresponding to their severity of COVID-19 pandemic, reaction to policy initiatives and more positive attitudes toward the importance of the vaccines.

### COVID-19 Vaccine Willingness Among 3 Jurisdictions

The survey period in Nepal was from August to November 2021, during which Nepal had experienced a downturn from the peak of COVID-19 pandemic. This period possibly boosted vaccine willingness during the peak related to perceived high-risk infection of COVID-19. In addition, the availability of COVID-19 vaccine was limited in Nepal with some donated by international organizations. It has been reported that limited resources affect an individual’s behavior.^22^ Our study suggested that vaccine willingness increased during the period when high incidence rates and limited stock of vaccines were observed. A fear of social neglect and discrimination may be another sociocultural reason that HCWs in Nepal showed a high interest for COVID-19 vaccinations.^23^

The survey period in Vietnam was from July to November 2021, when south Vietnam had the highest number of confirmed COVID-19 cases,^24^ and HCWs from other provinces volunteered to support them in fighting COVID-19. Considering the severity of the pandemic, as with Nepal HCWs, they strongly agreed with the statements about the effectiveness and importance of receiving COVID-19 vaccination to safeguard health, strengthen infection control in the working environment, achieve herd immunity, and mandatory vaccination, which resulted in a positive cooperation associated with the high COVID-19 vaccine willingness rate (>90%) observed in Vietnamese HCWs.^22^

The survey period in Hong Kong was from May to June 2021, which was right after the peak of the fourth wave of the COVID-19 pandemic, with much lower incidence and mortality rates than in Nepal and Vietnam. Hong Kong was relatively unscathed by the first 4 waves of the COVID-19 pandemic compared with most other places, and this was a possible reason for the low vaccine uptake and willingness rate (40%-50%). With less density of incidences, a contrasting phenomenon of high supply of COVID-19 vaccines seemed to have a comparatively lesser or even deleterious cooperation outcome associated with the vaccine willingness. In addition, policies against infection in Hong Kong, including border restrictions, quarantine and isolation, social distancing, and behavioral measures such as wearing masks, played an important role in driving the low incidence rate of COVID-19^25^ and may have created a sense of security that reduced the sense of need and acceptance of COVID-19 vaccination. One study using repeated surveys during the first and third waves of the COVID-19 outbreak in Hong Kong showed that the decreasing willingness to receive the COVID-19 vaccines might be associated with the increasing concerns about vaccine safety and increasing compliance with personal protection behaviors.^8^ However, vaccine acceptance may increase over time as additional information about risks and benefits of vaccine become more widely available. A study at the end of the fourth wave showed that the vaccine acceptance rate among the general population in Hong Kong had reached 67.8%.^26^

### Factors Associated With COVID-19 Vaccination Willingness

We observed that doctors were more willing to take COVID-19 vaccine than nurses. Doctors may have greater knowledge about the risk posed by SARS-CoV-2 and the coming pandemics than nurses, as well as the stronger belief in the efficacy and safety of the vaccination and a perception of personal susceptibility to COVID-19, as shown in a previous review that doctors were more likely than nurses to be vaccinated against pandemic influenza.^27^ We identified several social-demographic factors associated with vaccine willingness including older age, male, higher education level, and having seasonal influenza vaccination in the past year, which would increase the willingness to accept the COVID-19 vaccines, consistent with the findings of the previous reviews.^10,11,18,28^ Previous studies also demonstrated that older age and male gender were more likely to accept vaccination during the 2009 global influenza pandemic^27^ or prepandemic influenza vaccination at different WHO alert levels.^29^ We found that influenza vaccination history was a strong determinant of COVID-19 vaccine acceptance. These findings are consistent with studies from the UK,^30^ France,^31^ the US,^32^ and worldwide.^10,11,28^ This consistency may occur because those who have received influenza vaccination tend to pay more attention to the prevention of respiratory diseases and have more knowledge and positive attitudes regarding vaccination.^28^

### Strategies in Encouraging Vaccine Uptake

Among the 3 jurisdictions in our study, only Hong Kong had mandatory COVID-19 certification of showing either vaccination or negative test at the health care facility, which was expected to increase in the vaccination willingness.^33^ However, we found no obvious outcome in Hong Kong, possibly due to very low incident and mortality rates during the survey period. The full vaccination uptake rate in the general population increased from 6.6% during the fourth wave (between Nov 2020 to Apr 2021) to 77% with the average daily incident cases of more than 30 000 and the highest case fatality rate of 0.67% in the world during the fifth wave between Feb and Mar 2022.^17^ Therefore, the risk perception was possibly a factor associated with vaccine willingness when introducing mandatory COVID-19 certification. In addition, there was a significantly higher median score for “Don’t believe in the choice of the government” in Hong Kong than the other 2 jurisdictions. This was because Hong Kong just experienced social unrest before the COVID-19 pandemic in 2019, and vaccine hesitancy could be associated with depletion of trust in the government in monitoring the development and approval process of vaccines.^33,34^ Whereas common reasons for those unwilling to take COVID-19 vaccines in Nepal and Vietnam were inconvenient vaccination venues and time to get the vaccines. Thus, most of HCWs agreed a transportation allowance could be strategies to reduce vaccine hesitancy.

The availability of COVID-19 vaccine choices was believed to fulfill individual liberties and encourage vaccine willingness. Although 5 choices were available in Vietnam, the HCWs were provided with a strong recommendation of a certain brand by their senior management level. Therefore, they had similar attitudes toward “I don’t have choice for vaccine uptake” as those HCWs in Nepal in which only 1 choice was provided. By contrast, vaccine hesitancy increased due to inadequate information with choices,^8^ for example, confused by different vaccines and concerned about their safety and vaccine manufacturer benefits in both Hong Kong and Vietnam. Most participants preferred to wait for “better vaccine,” particularly in Hong Kong. Thus, transparency and timely information must go together with the choices to facilitate decision-making in vaccination.

With consideration of different health care systems, cultures and socioeconomic statuses, our findings were, however, consistent with arguments advocating for time off from work for vaccination as effective strategies to encourage vaccine uptake. Nepal and Vietnam showed more support for the immunity passport, which was consistent with a previous study that revealed “vaccine passports for overseas travel” as the strongest incentive for vaccination.^20^ Findings in this survey study may also be implicated to encourage the vaccine uptake in other jurisdictions of the Southeast Asian region.

### Limitations

This study has limitations. We observed a much higher prevalence of vaccine willingness among HCWs in Nepal and Vietnam than in Hong Kong, corresponding to their more positive attitudes and opinions toward the effectiveness, safety, and importance of the vaccines. However, the different vaccination rates would be mutually affected by pandemic waves, local number of daily confirmed and death cases, capacity of health care services, and relevant policies in different regions.^35^ We were unable to investigate the changes over time. Another limitation of the study is the varied survey periods during varied waves of the COVID-19 outbreak among the 3 jurisdictions. This limitation may bring challenges to direct comparison, but it enables data analysis in the context of different severities of the COVID-19 pandemic.

## Conclusions

Unwillingness to take COVID-19 vaccines exists among HCWs in Southeast Asia, especially in Hong Kong. In addition to sociodemographic characteristics and attitudes toward information and advice from government, a notable phenomenon was also observed for vaccine willingness in terms of availability of resources and severity of the COVID-19 pandemic. The desire for more comprehensive and updated information on vaccines including efficacy, adverse effects, and plan for the second generation of vaccination is an important factor associated with vaccine willingness. Thus, adequate facilitating measures such as vaccination leave and transportation subsidy are recommended to encourage more people to get vaccinated. To strengthen protection, explicit and transparent policy in terms of mandatory vaccination or vaccine passport is also necessary to boost the rate. A regular staff survey or staff engagement was important to incorporate their views into future directions of vaccine promotion.

Our findings may have utility in informing future public health or infection control policies and interventions, as well as strategies to promote or improve vaccine acceptance. Attitudes toward and willingness to accept vaccination among HCWs may provide an example for the general population, while it is also critical to investigate the changes in vaccine willingness over time.

## Notes

Supplement. **eMethods.** COVID-19 Vaccine Survey – HCWs
