# Supplementary material for: COVID-19 Vaccine Willingness and Related Factors Among Health Care Workers in 3 Southeast Asian Jurisdictions
Source: JAMA Netw Open. 2022 Aug 22;5(8):e2228061. doi: 10.1001/jamanetworkopen.2022.28061 (PMC9396363; doi:10.1001/jamanetworkopen.2022.28061)
Supplement: Supplement. — eMethods. COVID-19 Vaccine Survey – HCWs [file jamanetwopen-e2228061-s001.pdf]

Supplemental Online Content

Wong EL, Qiu H, Chien WT, et al. COVID-19 vaccine willingness and related factors among healthcare workers in 3 Southeast Asian jurisdictions. *JAMA Netw Open.* 2022;5(8):e2228061. doi:10.1001/jamanetworkopen.2022.28061

eMethods. COVID-19 Vaccine Survey – HCWs

This supplemental material has been provided by the authors to give readers additional information about their work.

© 2022 Wong ELY et al. *JAMA Network Open.*

eMethods. COVID-19 Vaccine Survey – HCWs

Session 1: Screening Questions

Q1.1 Do you agree to take part in this study?

◯ (Agree) Please continue to the next page. (1)

◯ (Not agree) Thank you for your time. This is the end of the survey. (2)

Q1.2 Are you Hong Kong residents aged 18 or above?

◯ Yes (1)

◯ No (2)

Q1.3 What is your employment status?

◯ Full time (1)

◯ Part time (2)

◯ Student (3)

◯ Unemployed (4)

◯ Retired (5)

Q1.4 What is your profession?

◯ Doctor (1)

◯ Nurse (2)

◯ None of the above (3)

© 2022 Wong ELY et al. *JAMA Network Open*.

Session 2

Q2.1 How likely do you think you would be diagnosed with COVID-19 in the next 12 months?

- ○ Most likely (1)
- ○ Likely (2)
- ○ Unlikely (3)
- ○ Most unlikely (4)

Q2.2 If you are infected with COVID-19, how serious do you think it will affect your health?

- ○ Very serious (1)
- ○ Serious (2)
- ○ Not serious (3)
- ○ Not serious at all (4)

Q2.3 How likely do you think the severity of the disease and the chance of developing complications would be reduced if a person is infected with COVID-19 even after receiving the vaccination?

- ○ Definitely (1)
- ○ Probably (2)
- ○ Probably not (3)
- ○ Definitely not (4)

© 2022 Wong ELY et al. *JAMA Network Open*.

Q2.4 The following are some statements about the COVID-19 vaccination. How much do you agree with these statements?

	Very agree (1)	Agree (2)	Disagree (3)	Very disagree (4)
Vaccination is the most effective way to combat COVID-19 at this stage	○	○	○	○
If more different types of vaccines are made available, it allows me to choose the most suitable vaccine	○	○	○	○
Vaccination advice should be made by Healthcare Professionals according to my physical condition	○	○	○	○
Several types of vaccine options are available, but the lack of comprehensive information makes me hesitate to uptake COVID-19 vaccination	○	○	○	○
The government should only provide one vaccine which most suitable for citizens	○	○	○	○

© 2022 Wong ELY et al. *JAMA Network Open*.

Q2.5 Given in good health, do you agree mandatory vaccination should be applied for the following categories of people?

	Very agree (1)	Agree (2)	Disagree (3)	Very Disagree (4)
Health care or residential care workers	○	○	○	○
Workers in service industry who have frequent contact with the public, such as drivers, catering and retail service, etc.	○	○	○	○
People who provide necessary community services, such as police, firefighters, etc.	○	○	○	○
Teachers	○	○	○	○

© 2022 Wong ELY et al. *JAMA Network Open*.

Session 3

Q3.1 Have you received COVID-19 vaccination?

- ◯ Never (1)
- ◯ Completed two doses (2)
- ◯ Received the first dose and will take the second dose (3)
- ◯ Received the first dose but will not take the second dose (4)

Q3.2 Are you planning to get the COVID-19 vaccine or make an appointment for vaccination in the future?

- ◯ Yes/ I have made an appointment for vaccination already (1)
- ◯ No (2)

Q3.3 The following are some reasons why people aren't getting the COVID-19 vaccine (or not getting the second dose of vaccine).

If score 0 represent "no effect", score 5 represent "medium effect", and score 10 represent "the greatest effect", how much do the following reasons affect your preference in vaccine uptake?

Please rate the following statements on a scale from 0 - 10, the higher the score, the greater the effect of the cause.

	0 1 2 3 4 5 6 7 8 9 10
I am aware of the instances of death or serious adverse effects following vaccination	
I am concerned of the vaccine manufacturer or its place of origin	
I want to wait for a better vaccine	
I am confused by different information of the vaccine	
I don't believe in the advice of the government	

© 2022 Wong ELY et al. *JAMA Network Open*.

Q3.4 Please rate the following statements on a scale from 0 - 10, the higher the score, the greater the effect of the cause.

	0 1 2 3 4 5 6 7 8 9 10
I don't believe the vaccine is effective to combat COVID-19	
The vaccination venues are inconvenient for me	
I think my physical condition is not allowed for vaccination	
Vaccine uptake is unnecessary for me	
I have no time to get the vaccine	
I don't have a choice in vaccine uptake	

Q3.5 The following are some strategies to encourage vaccine uptake among citizens.

If score 0 represent "no effect", score 5 represent "medium effect", and score 10 represent "the greatest effect", how much do the following strategies motivate you to get the COVID-19 vaccine?

Please rate the following statements on a scale from 0 - 10, the higher the score, the greater the motivation of getting the vaccine.

	0 1 2 3 4 5 6 7 8 9 10
To provide transportation allowance	
To authorise paid absence on the day of vaccination and the day after	
To implement "immunity passport" for travelling purpose	

Q3.6 It is assumed that "immunity passport" is implemented in Hong Kong to facilitate citizens in participation of certain public activities or access to certain premises.

If score 0 represent "no effect", score 5 represent "medium effect", and score 10 represent "the greatest effect", how much do following relaxation of social distancing measures motivate you to get the COVID-19 vaccine?

© 2022 Wong ELY et al. *JAMA Network Open*.

Please rate the following statements on a scale from 0 - 10, the higher the score, the greater the motivation of getting the vaccine.

	0 1 2 3 4 5 6 7 8 9 10
Religious gathering	
Visiting arrangements in hospitals and residential home	
Relaxation of mandatory quarantine	
Resumption of face-to-face class	
Easy access to some leisure and entertainment venues, such as restaurants, fitness rooms, sports fields, swimming pools, bars and karaoke box, etc.	

Q3.7 The following are the advices from different parties. If score 0 represent "no effect", score 5 represent "medium effect", and score 10 represent "the greatest effect", how much do the advice of the following parties alter your preference in vaccine uptake?

Please rate the following statements on a scale from 0 - 10, the higher the score, the greater the influence of the parties.

	0 1 2 3 4 5 6 7 8 9 10
Advice from doctor	
Advice from family members or relatives	
Advice from friends or colleagues	
Advice from employer	
Advice from the government	

© 2022 Wong ELY et al. *JAMA Network Open*.

Session 4

Q4.1 Have you received **<u>seasonal influenza vaccination</u>** last year?

- ◯ Yes (1)
- ◯ No (2)

Q4.2 How important is receiving COVID-19 vaccination to you in the following aspects?

	Very important (1)	Important (2)	Unimportant (3)	Very unimportant (4)
To safeguard your own health	◯	◯	◯	◯
To safeguard the health of family and friends	◯	◯	◯	◯
To strengthen infection control in the working environment	◯	◯	◯	◯
To achieve herd immunity	◯	◯	◯	◯

© 2022 Wong ELY et al. *JAMA Network Open*.

Session 5 – Demographic (voluntary)

Q5.1 What institute are you in?

- ◯ Public (1)
- ◯ Private / Non-governmental organization (2)
- ◯ Self-employed (3)

Q5.2 Which department are you in?

- ◯ Isolation ward/ Intensive care unit (1)
- ◯ General Ward (2)
- ◯ General Outpatient Clinic (3)
- ◯ Specialist Outpatient Clinic (4)
- ◯ A&E department (5)
- ◯ Nursing home, residential home or day centre (6)
- ◯ Outreach community service (7)
- ◯ School (8)
- ◯ Other (please specify) (9)

Q5.3 Gender

- ◯ Male (1)
- ◯ Female (2)

Q5.4 Age

- ◯ 18 - 29 years old (1)
- ◯ 30 - 39 years old (2)
- ◯ 40 - 49 years old (3)
- ◯ 50 - 59 years old (4)
- ◯ 60 - 69 years old (5)
- ◯ 70 years old or above (6)

© 2022 Wong ELY et al. *JAMA Network Open*.

Q5.5 Education level

- ◯ Secondary or below (1)
- ◯ Diploma/ Higher diploma (2)
- ◯ Bachelor's degree (3)
- ◯ Master's degree (4)
- ◯ Doctoral degree (5)

End of the survey

© 2022 Wong ELY et al. *JAMA Network Open*.
